# Anthropometric Parameters in Celiac Disease: A Review on the Different Evaluation Methods and Disease Effects

**DOI:** 10.1155/2019/4586963

**Published:** 2019-09-09

**Authors:** Allysson Costa, Gleisson A. P. Brito

**Affiliations:** Laboratory of Physiology and Developmental Biology, Federal University of Latin American Integration—UNILA, Foz do Iguaçu, Paraná, Brazil

## Abstract

This review compiled anthropometric data from 29 original articles, published between 1995 and 2015, corresponding to a total sample of 6368 celiac disease subjects. Body mass index was the main parameter for measuring anthropometry (82.1%), followed by body mass (78.6%), body fat (51.7%), bone mineral density and bone mineral content (46.4%), and fat-free mass (44.8%). The main evaluation method was dual x-ray absorptiometry (83.3%), followed by bioimpedance (16.6%), skinfold thickness (16.6%), and isotope dilution (5.5%). This compilation suggests that celiac disease patients without a gluten-free diet (WGFD) and celiac disease patients with a gluten-free diet (GFD) show a lower body mass than the control group, with inconclusive data about WGFD versus GFD. Body mass index is lower in WGFD and GFD compared to control group, and is lower in WGFD compared to GFD. We observed lower values of FM and FFM in WGFD and GFD versus the control group. No difference was found between WGFD versus GFD. BMD and BMC are lower in WGFD versus GFD and GFD versus the control group, with inconclusive data about WGFD versus GFD. The findings of this review suggest that celiac disease patients must be periodically evaluated through anthropometric parameters, since the pathology has the potential to modulate such values even in a gluten-free diet, with these variables reflecting their healthy status. In parallel, the screening of different anthropometric assessment methodologies can provide support for more accurate evaluations by scientists and clinical professionals who work with celiac disease patients.

## 1. Introduction

### 1.1. Celiac Disease

Celiac disease is a chronic autoimmune illness that manifests itself in individuals according to a genetic predisposition with environmental interaction [1]. It is characterized by an inflammatory condition as a consequence of the body's difficulty to process gluten proteins from wheat, barley, and rye [2–4]. Epidemiological research reveals a prevalence of 1 : 100 (1%) in the United States population, with a variation between 1 : 80 (1.25%) and 1 : 140 (0.71%) [3]. A previous review by Fasano et al. [5] estimated that celiac disease is one of the most frequent genetic disorders, affecting 0.5% to 1% of the world population. However, its diagnosis can be outdated, since its clinical presentation overlaps with other more common conditions.

Celiac disease manifests itself clinically in five ways: (1) classic: small bowel mucosal malabsorption, chronic diarrhea, abdominal distension, abdominal pain, weight loss, and flatulence; (2) atypical: the most common display of the disease, in which there is an absence of or few gastrointestinal symptoms, iron deficiency anemia, osteoporosis or osteopenia, infertility, and short stature; (3) silent: asymptomatic, with a casual, histological, or serological diagnosis; (4) latent: (A) individuals who are responsive to a gluten-free diet with a normal histology and elevated intraepithelial lymphocytes; (B) normal small bowel mucosa, without restriction to gluten, with subsequent development of celiac disease; (5) refractory: patients with celiac disease who do not respond to a gluten-free diet [4].

Each manifestation has its own characteristics, from gastrointestinal symptoms [6] to metabolic alterations [7, 8] and anthropometric changes, [9–11] mostly due to the unsatisfactory absorption of nutrients as a consequence of small bowel inflammation [6, 12]. The diagnosis of celiac disease is based on clinical manifestations and serological and histological laboratory tests from small bowel biopsies [13]. It is accepted that serological markers from tissue antitransglutaminase antibodies (TtG), immunoglobulin A (IgA), and antiendomysium are sensitive and specific to the initial celiac disease diagnosis [3, 14]. There is good evidence of a relationship between mucosal villi atrophies in the small intestine and the histopathological characteristics of the disease, and for this reason, a duodenal biopsy is recommended for diagnosis confirmation [14]. The only treatment for celiac disease is a gluten-free diet [2, 5], and patients with good adherence to it may present anthropometric values similar to healthy subjects [15]. However, other studies suggest that, compared to the control group, celiac patients with adherence to a gluten-free diet may still present decreased values in anthropometric parameters [9, 11, 16–18].

Considering the impact of celiac disease on metabolism and body composition, we now proceed to review these subjects and analyze data from experimental and epidemiological research.

### 1.2. Metabolic and Anthropometric Alterations in Celiac Disease Patients

The immunological process of celiac disease, triggered by gluten, leads to a chronic inflammatory response, resulting in lesions associated with atrophy in the small bowel mucosal villi [2], that results in unsatisfactory nutrient absorption, including fatty acids, iron, transferrin, glucose, electrolytes, vitamins, folic acid, and calcium [17, 19, 20]. As a consequence, short stature, muscular fatigue, iron deficiency anemia, hypovitaminosis (A, B12, D, K), muscle fatigue [6, 9, 17], bone mineral content reduction [10, 21], and osteoporosis, [22] among other intestinal symptoms [6], may occur. Furthermore, celiac disease patients oxidize more carbohydrates as an energy source, probably due to the insufficient absorption of nutrients by the small bowel mucosa [9]. In Bode et al.' study [16], celiac disease patients with good adherence to gluten-free diet, median age 42, compared to healthy subjects, had a lower body mass index, a lower body fat percentage, and a lower bone mineral content in the spine and forearms. González et al. [17] analyzed women with celiac disease, between 20 and 60 years, with or without a gluten-free diet, and compared them to a control group of women of the same age. Women with celiac disease, in both groups, had a lower body mass index and height compared to the control group, and women with celiac disease without a gluten-free diet presented a lower body mass index than those on a gluten-free diet, as well as a lower body fat and fat-free mass than the control group. Capristo et al. [9], when analyzing adult patients with celiac disease, with a mean age of 29.9 ± 7.6 years, found that those on a gluten-free diet presented a lower body mass, fat, and fat-free mass than the control group, with such parameters even lower in individuals without a gluten-free diet when compared to the control group. The research of Bardella et al. [18], with 71 celiac disease individuals without a gluten-free diet, between 17 to 58 years, verified that height in men, body mass, and body mass index in men and women were significantly lower than the control group. Brambilla et al. [11] studied 150 celiac disease children without a gluten-free diet and found lower values for body mass and body mass index when compared to the control groups.

Celiac disease can be effectively treated through a gluten-free diet [2, 5]. In the research by Barera et al. [23], individuals diagnosed with celiac disease revealed a lower body mass, fat mass, fat-free mass, and bone mineral content when compared to the control group, but after one year on a gluten-free diet, anthoropometric values were similar to healthy subjects. However, other evidence suggests that even with a gluten-free diet, patients with celiac disease can present lower anthropometric values, [9, 11, 16–18] in addition to nutritional deficiencies, such as lower levels of B6 and B12 vitamins, folic acid, iron, and transferrin [9, 24].

## 2. Methodology

For this review, original articles written in English were selected through the Google Scholar search engine, using the keywords: celiac disease, body composition, anthropometry, anthropometric, body mass, body mass index, lean mass, fat mass, fat-free mass, bone mineral content, and bone mineral density. The articles were published between 1995 and 2015, covering a period of 20 years.

The inclusion criterion used was the presence of anthropometric data in celiac disease patients. The search returned 29 original articles, corresponding to a total sample of 6368 subjects with celiac disease. All data were included for review and discussion.

Data were compiled according to evaluated anthropometric parameters, the sex and age of the sample, and the methodology undertaken. Data from the anthropometric parameters were distributed in 3 groups: celiac disease patients without a gluten-free diet (WGFD), celiac disease patients with a gluten-free diet (GFD), and the control group.

## 3. Results

### 3.1. Measured Parameters and Sample Characteristics

In the reviewed studies, an analysis of body mass index (BMI) was present in 82.1%, body mass (BM) in 78.6%, fat mass in 54.7%, fat-free mass in 44.8%, and bone mineral density (BMD) and bone mineral content (BMC) in 46.4% of the studies (Table 1).

Table 2 compiles the sample data of age and sex and the presence of control groups in the reviewed studies. A prevalence of adults, 3197, in relation to children and adolescents, 443, was verified in the proportion of 7 : 1, with more female adult (52.9%) than male adult (47.1%) patients. In children and adolescents, we are also found more females (52.2%) than males (47.8%). In the control groups adults were more prevalent, 70.5%, with children and adolescents representing 29.5%.

### 3.2. Anthropometric Measurement Methods

In order to evaluate anthropometry, the studies used different methodologies, including digital scales and weight balances for body mass, wall, and infant stadiometers for height measurement, plicometer, bioimpedance, isotope dilution, and dual-energy x-ray absorptiometry (DXA) for fat mass and fat-free mass, and DXA for measuring bone mineral density and bone mineral content. The body mass index (BMI) was mathematically defined by the weight/height ratio, with the formula BMI = weight (kg)/height^2^ (m).

In Table 3, the frequency of different anthropometric measurement methods is compiled. DXA is present in 83.3%, bioimpedance in 16.6%, skinfold thickness in 16.6%, and isotope dilution in 5.5% of the studies. Moreover, the most used DXA system was Lunar DPX, present in 9 studies, followed by Lunar Prodigy and Hologic Delphy, present in 2 studies each, and Lunar DPA was used in 1 study.

### 3.3. Body Mass in Patients with Celiac Disease

The WGFD group presented a lower BM when compared to the GFD group, in 50% of the studies. This parameter was not different in the other 50% of studies. However, when compared to the control group, WGFD presented a lower BM in 100% of the reviewed studies. When GFD was compared to the control group, 66% of the studies showed lower values, while 33.35% revealed no difference between these groups (Table 4). Seven studies were not included in the BM table because they did not discriminate between these data.

### 3.4. Body Mass Index in Patients with Celiac Disease

When WGFD versus GFD and WGFD versus the control group were compared, 71.4% of studies showed a lower BMI, while 28.6% presented no difference between the groups. However, GFD compared to the control group presented a lower BMI in 60% of the studies, and no difference in 40% of them (Table 5). Eight articles did not discriminate this parameter between the groups and were not included in Table 5. Passananti et al. compared WGFD to GFD in two time periods, at two and five years, and found different results, which were included separately in Table 5.

### 3.5. Fat Mass and Fat-Free Mass in Patients with Celiac Disease

FM data are compiled in Table 6. When compared to GFD and to the control group, 100% of the studies presented a lower FM in the WGFD group. When GFD was compared to the control group, 50% of studies found no difference, 41.7% showed a lower FM, and 8.3% a higher FM.

Table 7 presents the compiled FFM data. Compared to GFD, WGFD showed a lower FFM in 66% of studies, and there was no difference in 33.3% of them. However, WGFD versus control subjects presented lower FFM values in 100% of the studies. When compared to the control group, 36.4% of studies showed a lower FFM in GFD, while 63.6% found no difference.

Four articles, one for FM and three for FFM, did not discriminate data relative to this parameter and were not included in Tables 6 and 7.

### 3.6. Bone Mineral Density and Bone Mineral Content

When WGFD was compared to the control group, it was found to have a lower BMD and BMC in 90% of the studies. In 66.5% of the studies, GFD presented a lower BMD and BMC versus control patients, and there was no difference in this comparison in 33.3% of the studies. There were not enough data to compare WGFD and GFD (Table 8) in relation to BMD and BMC.

## 4. Discussion

The anthropometric assessment is an important variable to understand human metabolism in different health conditions, [47, 48] including celiac disease. The data presented in our sample of studies, there is a greater level of verification in the body mass index (BMI) 82.1%, and body mass (BM) 78.6%. These techniques are complementary, can be easily accessed, and have a low cost. More complex and expensive variables were used in smaller proportions in anthropometric studies involving patients with celiac disease: fat mass (FM) 53.6%, fat-free mass (FFM) 44.8%, bone mineral density (BMD) 46.4%, and bone mineral content (BMC) 9%. Despite the usefulness of BMI in the classification of subjects, [49] other variables like fat mass, fat-free mass, bone mineral density, and bone mineral content enable a more comprehensive analysis of anthropometry. Future studies on these topics needs to consider the complexity effects of celiac disease in anthropometric parameters to provide for an appropriate selection of assessment methods.

The reviewed studies presented a higher proportion of adult patients with celiac disease than children and adolescents, being 7 : 1, respectively, and a greater participation of females (53.3%) than males (46.7%). A recent review of ninety-six studies published between 1991 and 2016, indicated a higher prevalence of celiac disease in females than males (0.6% versus 0.4%) and in children than adults (0.9% versus 0.5%). [50] Our data suggests that more research with children and adolescents is necessary to establish a better understanding of the anthropometric impacts as well as the growth and development of celiac disease.

Regarding the anthropometry measurement methods, a higher prevalence of DXA was observed, at 83.3% which, despite its high cost, has the main advantage of simultaneously analyzing fat mass, fat-free mass, and bone mineral content; moreover, it is considered the gold standard for body composition measurements. [47, 48, 51, 52] In this review, Lunar DPX was the most used DXA device, followed by Lunar Prodigy, Hologic Delphy, and Lunar DPA. A previous review from Marinangeli and Kassis [53] about DXA comparative studies indicate that there are inter- and intradevices differences, with under or overestimated values, which may be explain by factors like beam type, gender sample, and scan speed. However, quality-control procedures may help the correct identification of changes in body composition parameters [53].

Bioimpedance, with a frequency of 16.6%, depends on many factors to obtain validity and precision, such as the type of apparatus, researcher handling, room temperature, and the preparation of subjects in relation to feeding, hydration, physical exercise routine, and the consumption of alcohol and medicine [54]. These limitations may explain the lower utilization of bioimpedance in these studies. The skinfold thickness evaluation is one of the most popular research methods because of its low cost and simplicity, and there is a good relation between subcutaneous fat and total fat mass [47, 55]. However, its frequency of use in these studies was low, a factor that can be explained by its limitation to predict bone mineral density and bone mineral content, being variables present in a larger proportion of the reviewed studies. Isotope dilution was the least utilized method, at 5.5%. This method verifies the deuterium, oxygen eighteen, and tritium concentration, thus determining the total water, fat mass, and fat-free mass for the whole body [56]. Despite having good precision, the technique is very difficult to analyze, [47] being a possible cause for its restricted use. We credit the greater preference for the DEXA method among others to two factors: (1) the possibility of accessing many variables simultaneously, allowing for more complete and faster analyses; (2) the higher incidence of bone mineral density and bone mineral content in the reviewed methodologies, which are variables easily measured by DEXA devices.

Considering the lack of studies with two or more body composition assessment methodologies, we cannot do a comparative evaluation between them, since it is not possible to claim that the result is a consequence of the selected method or of group characteristics. However, previous research suggests that skinfold thickness and bioimpedance seems to provide underestimated results for body fat when compared to DXA devices [57, 58].

Body mass was evaluated in 80.7% of the reviewed studies. All studies that evaluated body mass in WGFD patients and most of the GFD studies revealed significantly lower levels of this parameter when compared to the control group, corroborating previous findings [9–11, 18, 23, 27, 28, 32, 36]. Although many publications present a gluten-free diet as a body mass promoter [11, 20, 23, 30, 40], our review found that in half of the studies, there was no significant difference between WGFD and GFD groups.

The majority of the studies indicated that WGFD presented lower values of BMI than control subjects, in accordance with previous observations [27, 36, 40, 42, 59]. However, the majority of studies showed higher BMI values in WGFD compared to GFD, suggesting that BMI seems to be influenced by a gluten-free diet [11, 20, 21, 30, 41]. However, most studies also demonstrated that WGFD have lower values of BMI when compared to the control group, suggesting that the gluten-free diet may not be able to normalize this parameter [11, 16, 32, 39]. In the work of West et al. [35], which proposed a categorization of samples thorough BMI, a greater prevalence of WGFDs in the underweight classification was verified. In addition, Kabbani et al. [42] observed a significant probability of WGFD and GFD patients to be in the underweight classification in relation to the overweight and obesity classifications.

The mensuration of fat mass and fat-free mass in patients with celiac disease constitutes an important parameter to investigate the effects of this disease on anthropometric and metabolic functioning. Moreover, the increased body mass found in GFD subjects seems to be essentially related to an increase in fat mass [9, 20, 30, 36]. The studies compiled indicated lower values of FM in WGFD compared to GFD and the control group, corroborating with previous publications [9, 10, 16, 18, 23, 27, 28, 30, 32, 36, 40]. Although all the publications revealed a rise of FM in GFD patients, only half of these studies showed the same values when GFD patients are compared to the control group [23, 28, 30, 36, 43, 60].

In the majority of publications, GFD presented lower values of FFM than the control group, suggesting that a gluten-free diet may not be able to normalize FFM values in relation to healthy people [9, 10, 17, 18, 30, 32, 36].

The analysis of bone mineral density and bone mineral content revealed that the majority of publications found significantly lower levels of these variables in WGFD than in control groups [10, 17, 18, 21, 23, 29, 30, 32, 37, 40, 44]. A few publications suggest that a gluten-free diet significantly promotes bone mineral density and bone mineral content [21, 29, 30] with some articles indicating that celiac disease patients submitted to a one year gluten-free diet can present normal values of these parameters [10, 23, 44]. However, most of the reviewed studies showed lower mineral density and bone mineral content values in GFD patients compared to control groups [17, 21, 25, 29, 30, 40, 44].

Our limitations are similar to those observed by Bardella et al., which includes the difficulty in comparing previously published anthropometry and nutritional data in patients with celiac disease. Data ambiguity and the dependence on other variables such as age at diagnosis, symptom duration before diagnosis, and the presence or absence of unsatisfactory nutrient absorption constitutes important factors of interference.

## 5. Conclusion

Celiac disease significantly changes anthropometric parameters, which can be improved by a gluten-free diet. Despite this, celiac disease patients may not improve anthropometric variables to values similar in healthy people.

This review demonstrated that WGFD compared to the control group showed lower values in all anthropometric variables. GFD compared to WGFD presented higher values in BMI, FM, and FFM, and there was inconclusive data about BM, BMD, and BMC. GFD patients did not present different FFM values from control.

Anthropometric parameters that were more utilized included body mass index and body mass, followed by fat mass, bone mineral density and bone mineral content, and fat-free mass, which were most measured by dual-energy x-ray absorptiometry (DXA), followed by bioimpedance, skinfold thickness, and isotope dilution.

The findings of this review suggest that celiac disease patients must be periodically evaluated through anthropometric parameters, since the pathology has the potential to modulate such values even in a gluten-free diet, with these variables reflecting their healthy status. In parallel, the screening of different anthropometric assessment methodologies can provide support for more accurate evaluations by scientists and clinical professionals who work with celiac disease patients.

## Figures and Tables

**Table 1:** Anthropometric parameters measured in studies with celiac patients.

Scientific publication	BM	BMI	BMC	BF	FFM
			BMD		
Bode et al. [25]	X	X	X	X	
Bardella et al. [26]	X	X			
Gonzalez et al. [27]	X	X	X	X	X
Rea et al. [28]	X	X	X	X	X
Mautalen et al. [29]			X		
Smecuol et al. [30]	X		X	X	X
Dickey and Bodkin [31]	X	X			
De Lorenzo et al. [32]	X	X	X	X	X
Bardella et al. [18]	X	X	X	X	X
Barera et al. [23]	X	X	X	X	X
Capristo et al. [9]	X			X	X
Fabiani et al. [33]					
Carbone et al. [10]	X		X	X	X
Zipser et al. [34]		X			
West et al. [35]		X			
Capristo et al. [36]	X	X		X	X
Dickey and Kearney [37]	X	X	X		
West et al. [38]		X			
Capristo et al. [20]	X	X		X	X
Cheng et al. [39]	X	X			
Duerksen and Leslie [40]	X	X	X	X	
Reilly et al. [41]		X			
Kabanni et al. [42]		X			
Passananti et al. [21]	X	X	X		
Brambilla et al. [11]	X	X			
Valente [43]	X	X		X	X
Kurpaa et al. [44]	X	X	X		
Silva et al. [45]	X	X		X	X
Churruca et al. [46]	X			X	X
Totais de um mesmo parâmetro	78.6%	82.1%	46.4%	51.7%	44.8%

BM, Body mass; BMI, body mass index; BMD, bone mineral density; BMC, bone mineral content; FM, fat mass; FFM, fat-free mass.

**Table 2:** Sample characteristics in studies with celiac patients.

Scientific publication	<18 years		>18 years		Control
	M	F	M	F	
Bode et al. [25]			7	15	No
Bardella et al. [26]			31	127	No
Gonzalez et al. [27]				32	85
Rea et al. [28]	8	15			No
Mautalen et al. [29]			5	9	No
Smecuol et al. [30]			1	24	No
Dickey and Bodkin [31]			35	15	No
Lorenzo et al. [32]	12	31			30
Bardella et al. [18]	14	15	8	15	52
Barera et al. [23]			20	51	142
Capristo et al. [9]			16	23	63
Fabiani et al. [33]			26	18	No
Carbone et al. [10]	15	33			41
Zipser et al. [34]			748	248	No
West et al. [35]		2649^*∗*^	17925		
Capristo et al. [36]				18	22
Dickey and Kearney [37]			114	257	No
West et al. [38]			57	30	No
Capristo et al. [20]			17	9	No
Cheng et al. [39]			248	121	No
Duerksen and Leslie [40]			6	37	233
Reilly et al. [41]	60	82			No
Kabanni et al. [42]			166	513	No
Passananti et al. [21]				95	No
Brambilla et al. [11]	103	47			288
Valente [43]			8	12	39
Kurpaa et al. [44]			40^*∗*^	No	
Silva et al. [45]	23	8			31
Churruca et al. [46]				54	No
Totais	212	231	1513	1725	18975

The data was separated in children and adolescents (<18 years) and adults (>18 years), subdivided by sex, male (M) and female (F). Control: the presence of a control groups. ^*∗*^Sex data not informed. These values were not included in stratification by sex.

**Table 3:** Anthropometric measurement methods in celiac patients studies.

Scientific publication	ST	BIA	DXA	ID	DXA devices
Bode et al. [25]	X		X		Lunar DPA
Gonzalez et al. [27]			X		Lunar DPX
Rea et al. [28]	X		X		Lunar DPX
Smecuol et al. [30]			X		Lunar DPX
Lorenzo et al. [32]	X	X	X		Lunar DPX
Bardella et al. [18]			X		Hologic
Barera et al. [23]			X		Lunar DPX
Capristo et al. [9]				X	
Carbone et al. [10]			X		Lunar DPX
Capristo et al. [36]			X		Lunar DPX
Dickey and Kearney [37]			X		Not shown
Capristo et al. [20]			X		Lunar prodigy
Duerksen and Leslie [40]			X		Lunar DPX
Passananti et al. [21]			X		Hologic
Valente [43]			X		Lunar DPX
Kurpaa et al. [44]			X		Lunar prodigy
Silva et al. [45]		X			
Churruca et al. [46]		X			
Totais	16.6%	16.6%	83.3%	5.5%	

ST, skinfold thickness; BIA, bioimpedance; DXA, dual-energy X-ray absorptiometry; ID, isotope dilution.

**Table 4:** Body mass of celiac patients with and without gluten-free diet.

Scientific publication	WGFD versus GFD	WGFD versus control	GFD versus control
Gonzalez et al. [27]	Lower	Lower	Lower
Rea et al. [28]	Equal	Lower	Lower
Smecuol et al. [30]	Lower		
Lorenzo et al. [32]			Equal
Bardella et al. [18]			Lower
Barera et al. [23]		Lower	Equal
Capristo et al. [9]	Lower	Lower	Lower
Carbone et al. [10]		Lower	Lower
Capristo et al. [36]	Equal	Lower	Lower
Capristo et al. [20]		Lower	Lower
Duerksen and Leslie [40]		Lower	
Brambilla et al. [11]			Lower
Valente [43]			Equal
Kurpaa et al. [44]	Equal		
Silva et al. [45]			Equal

**Table 5:** Body mass index of celiac patients with and without gluten-free diet.

Scientific publication	WGFD versus GFD	WGFD versus control	GFD versus control
Bode et al. [25]			Lower
Bardella et al. [26]		Lower	Lower
Gonzalez et al. [27]	Lower	Lower	Lower
Rea et al. [28]		Equal	Equal
Bardella et al. [18]			Lower
Barera et al. [23]		Equal	Equal
Capristo et al. [36]	Lower	Lower	Lower
Capristo et al. [20]	Lower		
Duerksen and Leslie [40]		Equal	
Kabanni et al. [42]	Lower	Lower	
Passananti et al. [21]	Equal		
Passananti et al. [21]	Lower		
Brambilla et al. [11]			Lower
Valente [43]			Equal
Kurpaa et al. [44]	Equal		
Silva et al. [45]			Equal

**Table 6:** Fat mass of celiac patients with and without gluten-free diet.

Scientific publication	WGFD versus GFD	WGFD versus control	GFD versus control
Bode et al. [25]			Lower
Gonzalez et al. [27]	Lower	Lower	Lower
Rea et al. [28]		Lower	Equal
Smecuol et al. [30]	Lower	Lower	Equal
Lorenzo et al. [32]			Lower
Bardella et al. [18]		Lower	Lower
Barera et al. [23]		Lower	Equal
Capristo et al. [9]			Higher
Carbone et al. [10]			Lower
Capristo et al. [36]		Lower	Equal
Capristo et al. [20]	Lower		
Duerksen and Leslie [40]		Lower	
Valente [43]			Equal
Silva et al. [45]			Equal

**Table 7:** Free fat mass of celiac patients with and without gluten-free diet.

Scientific publication	WGFD versus GFD	WGFD versus control	GFD versus control
Gonzalez et al. [27]	Lower	Lower	Lower
Rea et al. [28]		Lower	Equal
Smecuol et al. [30]		Lower	Lower
Lorenzo et al. [32]			Lower
Bardella et al. [18]		Lower	Lower
Barera et al. [23]		Lower	Equal
Capristo et al. [9]	Equal	Lower	Lower
Carbone et al. [10]			Lower
Capristo et al. [36]		Equal	Lower
Capristo et al. [20]	Lower		
Valente [43]			Equal
Silva et al. [45]			Equal

**Table 8:** Bone mineral density and bone mineral content of celiac patients with and without gluten-free diet results.

Scientific publication	WGFD versus GFD	WGFD versus control	GFD versus control
Bode et al. [25]			Lower
Gonzalez et al. [27]	Lower	Lower	Lower
Mautalen et al. [29]		Lower	Lower
Smecuol et al. [30]		Lower	Lower
De Lorenzo et al. [32]		Lower	
Bardella et al. [18]		Lower	
Barera et al. [23]		Lower	Equal
Carbone et al. [10]		Lower	Equal
Dickey and Kearney [37]		Lower	
Duerksen and Leslie [40]		Lower	Lower
Passananti et al. [21]		Lower	Lower
Kurpaa et al. [44]		Equal	Equal

## Abbreviations

BMI: Body mass index
BM: Body mass
BF: Body fat
BMD: Bone mineral density
BMC: Bone mineral content
FFM: Fat-free mass
WGFD: Celiac disease patients without a gluten-free diet
GFD: Celiac disease patients with a gluten-free diet
TtG: Antitransglutaminase antibodies
IgA: Immunoglobulin A
DXA: Dual-energy X-ray
ST: Skinfold thickness
BIA: Bioimpedance
ID: Isotope dilution.
